# Why Does a Cardiologist Believe in a Therapy? The Role of Intuitiveness and Understanding the Mechanism

**DOI:** 10.1161/CIRCOUTCOMES.123.010664

**Published:** 2024-02-16

**Authors:** Michael J. Foley, Christopher A. Rajkumar, Fiyyaz Ahmed-Jushuf, Alexandra N. Nowbar, Florentina Simader, Olamide Bello, Rasha Al-Lamee

**Affiliations:** 1 National Heart and Lung Institute, Imperial College London, United Kingdom (M.J.F., C.A.R., F.A.-J., A.N.N., F.S., O.B., R.A.-L.).; 2 Imperial College Healthcare NHS Trust, London, United Kingdom (M.J.F., C.A.R., R.A.-L.).

**Keywords:** cardiologists, myocardial infarction, percutaneous coronary intervention, randomized controlled trials, United Kingdom

It is widely accepted that a medical intervention should demonstrate efficacy in the setting of a randomized controlled trial (RCT) before progressing to routine clinical use. For interventions designed to improve symptoms, we believe that the bar should be even higher, necessitating that the effect of a therapy be compared with placebo in a double-blind trial. In reality, many interventions that are routinely used and enthusiastically endorsed within cardiology do not have these supportive data. Why, then, do we persist with these therapies?

Unlike pharmacotherapy, where the mechanism is not visualizable and, therefore, often less tangible to a clinician, procedural interventions in cardiology have an observable, physical effect, typically imparted personally by the hands of the operator. How a procedure is beneficial to a patient may, therefore, seem intuitive, or obvious, to a cardiologist. The physical effect that is imparted by an intervention may align precisely with the cardiologists’ preexisting paradigm of the disease state borne by the patient and how that disease can be altered.

Perhaps the most mechanistically intuitive procedure to a contemporary cardiologist is the primary percutaneous coronary intervention for ST-segment–elevation myocardial infarction. There are unequivocal randomized data supporting its use but these arrived after a mechanistically driven belief that opening an acutely occluded coronary artery would improve outcomes for a patient.^1,2^ Still other highly intuitive interventions in cardiology, for example, permanent pacing in complete atrioventricular block and surgical aortic valve replacement in severe aortic stenosis, have never been subjected to a randomized trial and, as there is clearly no community equipoise, likely never will be. The efficacy of these interventions is felt to be obvious and to test this would be unnecessary, even unethical.

In contrast, occasionally therapies within cardiology are found to be effective in a randomized setting before we have a mechanistically plausible explanation as to why they might work. A contemporary example is the coronary sinus reducer. This is an hourglass-shaped wire-mesh device, designed to narrow the coronary sinus to a 3 mm lumen. The coronary sinus reducer was found to improve angina significantly when compared with placebo.^3^ However, although coronary sinus obstruction in the setting of myocardial infarction has been demonstrated to improve myocardial salvage and increase subendocardial perfusion, how a fixed luminal reduction in the coronary sinus might improve angina in a stable setting remains unknown.^4,5^ In theory, the robust level of evidence provided by a placebo-controlled RCT should be convincing, but it clearly has not been for the majority of cardiologists, and indeed the authors of clinical guidelines.^6^

We aimed to explore the association between the intuitiveness of a therapy’s mechanism and the perceived efficacy of that therapy by conducting an online survey among cardiologists. We hypothesized that procedures with a high level of mechanistic intuitiveness would be perceived to be highly effective. This association may go some way to explaining the apparent paradox of intuitive therapies persisting in clinical practice without randomized or placebo-controlled data, with abstract therapies not being widely used despite having data to support their use.

We conducted an online survey, distributed via email invitation, to 108 cardiologists at 6 centers in the United Kingdom. Respondents were asked to assess 25 different procedures within cardiology. For each therapy, they were asked to grade firstly how intuitive, or obvious, the mechanism of action was for a specific indication and secondly, their opinion on how effective the therapy was, giving each domain a score between 1 and 5 (with 5 being the most intuitive or the most effective, respectively). The interventions were chosen by a panel of cardiologists with a range of cardiology experience, aiming to represent interventions across multiple interventional cardiac subspecialties, with a broad range of levels of evidence to support their use. All these interventions have either currently, or previously, been licensed for use within the United Kingdom in routine clinical practice or human research.

The survey was responded to by 59 cardiologists (response rate, 54.6%). Twenty-six (44.1%) were consultants and 33 (55.9%) were in cardiology training. 18 (30.5%) were not subspecialized, 17 (28.8%) were subspecialized in coronary intervention, 11 in cardiac electrophysiology (18.6%), 10 in cardiovascular imaging (16.9%), and 2 (3.4%) in heart failure. The mean intuitive mechanism score for the interventions was 3.0±0.22. The mean subjective efficacy score for the interventions was 3.42±0.23. These scores were strongly correlated (*R*=0.945; Figure 1).

**Figure 1.**
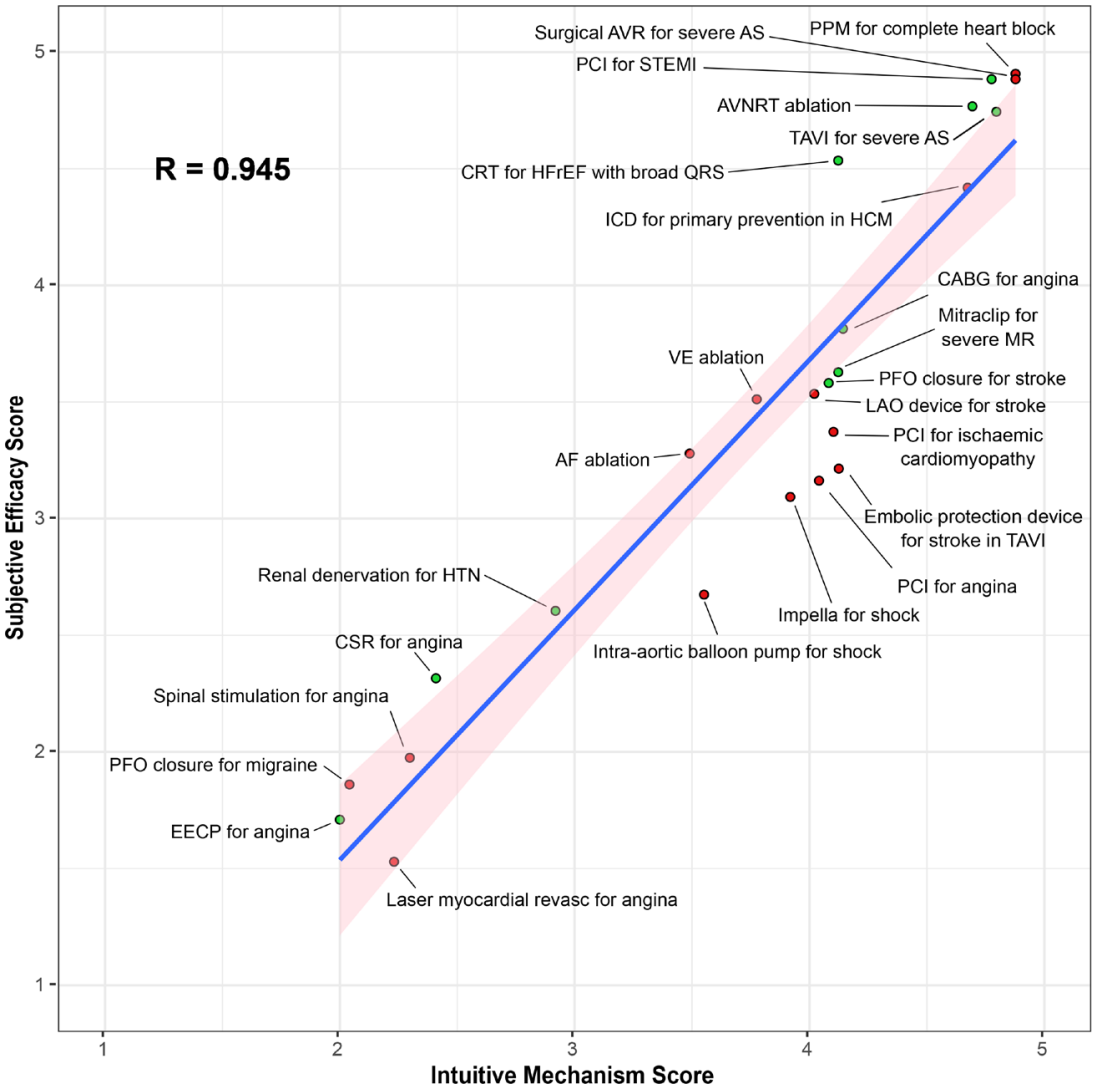
**The relationship between intuitive mechanism score and subjective efficacy score for the interventions in the study.** The blue line represents the trend line for the relationship and the pink shaded area represents the 95% CI. Points are shaded green if the intervention has been tested in at least 1 phase 3 randomized controlled trial, which was positive in favor of the intervention for the prespecified primary end point. Superiority trial designs and a placebo-control group if the measured outcome was subjective were required. If these data are not available, the points are shaded red. This survey was conducted in 2021 that predated the recent publication of ORBITA-2 showing placebo-controlled evidence of angina relief with PCI. AF indicates atrial fibrillation; AS, aortic stenosis; AVNRT, AV nodal reentrant tachycardia; AVR, aortic valve replacement; CABG, coronary artery bypass grafts; CRT, cardiac resynchronization therapy; CSR, coronary sinus reducer; EECP, enhanced external counterpulzation; HCM, hypertrophic cardiomyopathy; HFrEF, heart failure with reduced ejection fraction; HTN, hypertension; ICD, implantable cardioverter defibrillator; LAO, left atrial appendage; MR, mitral regurgitation; PCI, percutaneous coronary intervention; PFO, patent foramen ovale; PPM, permanent pacemaker; Revasc, revascularization; STEMI, ST-segment–elevation myocardial infarction; TAVI, transcatheter aortic valve implantation; and VE, ventricular ectopic.

In this study, we have found that among cardiologists, the intuitiveness of a therapy’s mechanism of action is strongly associated with the subjective view of its efficacy. This may be because the most mechanistically intuitive therapies truly are the most effective. However, in many cases, this has not been borne out in RCTs with appropriate control groups. Alternatively, this may reflect the value that cardiologists place on the mechanistic plausibility of a therapy over and above randomized data.

There are some therapies, which are likely to never be tested in an RCT against a suitable control. In most cases, the belief in the efficacy of the therapy is sufficiently strong that it is felt either unnecessary to test the therapy or unethical deny it to the control arm. These trials would represent the so-called randomized trial of the parachute.^7^ One example of this is surgical aortic valve replacement in severe aortic stenosis. Our firm belief in the efficacy of this therapy may be supported by diverse influences—historical, unrandomized, and observational data noting an improvement in survival in operated patients, our own clinical, anecdotal experience of the success of the therapy, and perhaps, our mechanistic understanding of the disease and the impact on this disease of the therapy. We should ask ourselves—would we accept this level of evidence for a new drug? The challenge as clinical trialists is to identify which interventions are truly parachutes and, in the broader context of contemporary medical therapy, modern lifestyles, and competing procedures, is our patient truly at the altitude that they perhaps were at the procedure’s conception?

PCI instable angina was developed as an antianginal procedure but a belief in its prognostic importance began to develop in the years following its conception. This may be because the stenosis and the associated ischemia were felt to be the cause of future adverse events or perhaps because the invasive management of acute coronary syndrome had trained us to believe that all stenoses must be treated. Our modern view of PCI as an antianginal procedure only has evolved from repeated randomized data showing no benefit in hard outcomes with PCI and also with the evolution of our mechanistic understanding of the underlying cause of myocardial infarction. We now recognize that plaque composition and stability are more influential on the risk of myocardial infarction than the tight anatomic stenosis causing ischemia downstream.^8^ Unlike PCI, less mechanistically plausible interventions may be dismissed without a trial ever being conducted, or after the first negative result.

What makes an intervention intuitive is complex and may be related to analogies we can draw with our everyday experience. The paradigmatic example of this is William Harvey’s demonstration of the heart as a pump at the center of a circulatory system coming after the first conception of the mechanical water pump, which may have made this inference more readily apparent.^9^ Our everyday experience in cardiology may inform our contemporary model of a condition and the role that a procedure can play in treating it. This may be passively learned from numerous sources. In modern cardiology practice, making an intervention seem intuitive may even be the deliberate sales strategy of the company who markets the device.

As cardiologists, we should be aware of these cognitive biases when appraising a therapy. Perhaps we are ready to endorse intuitive therapies and, therefore, set a lower bar for efficacy in RCTs. If the benefit of an intervention is made to seem mechanistically obvious, we may go further still and declare the intervention a parachute. There is a continuous relationship between mechanistic intuitiveness and our acceptance of a therapy with some more supported by a clear mechanisms and others by randomized data. Our strongest views may be on therapies that have neither or both (Figure 2). Conversely, we may also dismiss effective therapies, which we do not yet fully understand. This interrelationship requires that alongside RCTs to determine a treatment effect, we also conduct mechanistic studies to define the physiological impact of a therapy.

**Figure 2.**
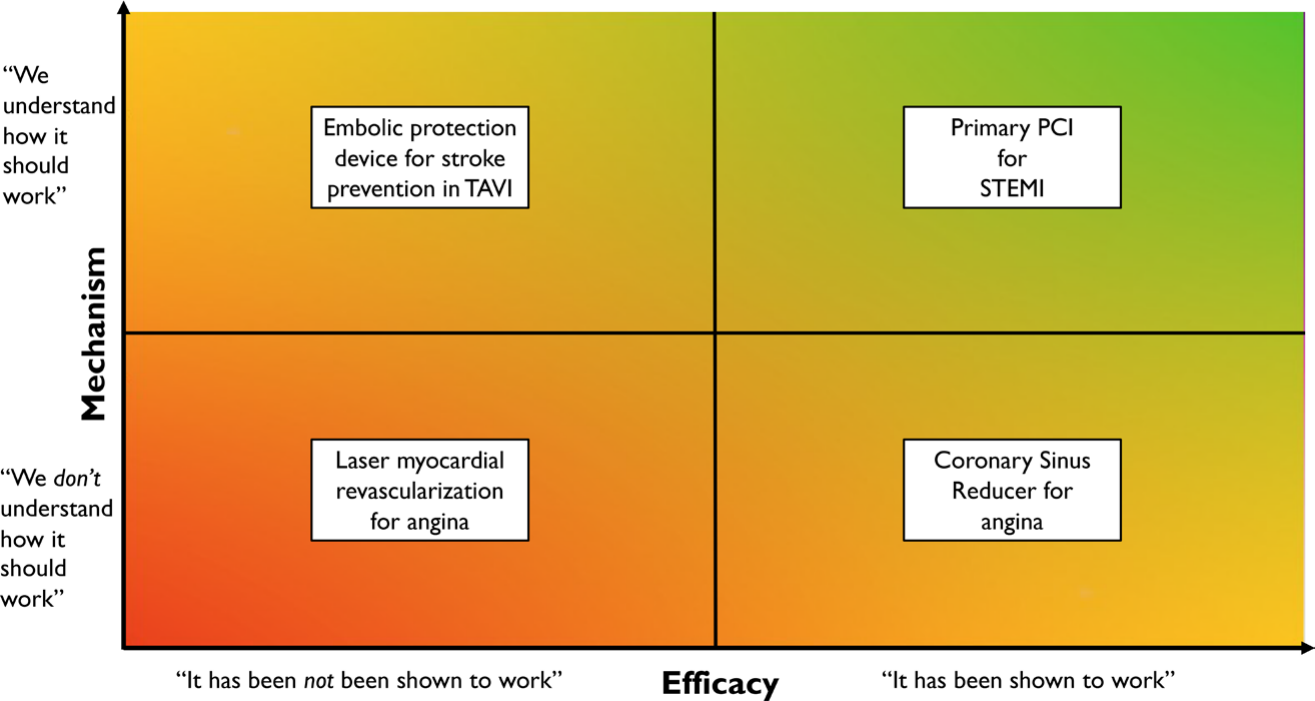
**A graphic representation of the postulated interaction between mechanistic intuitiveness and efficacy.** The green upper right-hand panel represents interventions, which are both mechanistically intuitive and have been demonstrated to work in an RCT with an appropriate control. An example of such a therapy is primary percutaneous coronary intervention (PCI) in ST-segment–elevation myocardial infarction (STEMI). The red lower left-hand panel represents unintuitive therapies which have not been demonstrated to work. These therapies have been abandoned in most cases, an example being laser myocardial revascularization for angina. The amber panels represent therapies where the mechanistic intuitiveness of the therapy and the randomized data to support it are discordant. Contemporary examples are embolic protection devices for prevention of stroke in transcatheter aortic valve implantation (TAVI) and Coronary Sinus Reducer for angina.

## ARTICLE INFORMATION

### Source of Funding

M.J. Foley, C.A. Rajkumar, and F. Ahmed-Jushuf are funded by the Medical Research Council (MR/V001620/1, MR/S021108/1, and MR/W000520/1). Dr Al-Lamee is funded by the British Heart Foundation (FS/ICRF/22/26051).

### Disclosures

M.J. Foley, C.A. Rajkumar, and Dr Al-Lamee have received speakers’ honoraria from Philips Volcano and Menarini Pharmaceuticals. Dr Simader has received speaker’s honoraria from Servier Pharmaceuticals. The other authors report no conflicts.

## References

[1] Keeley EC Boura JA Grines CL. Primary angioplasty versus intravenous thrombolytic therapy for acute myocardial infarction: a quantitative review of 23 randomised trials. Lancet. 2003;361:13–20. doi: 10.1016/S0140-6736(03)12113-7 12517460 10.1016/S0140-6736(03)12113-7

[2] Rentrop KP Blanke H Karsch KR Wiegand V Köstering H Oster H Leitz K. Acute myocardial infarction: intracoronary application of nitroglycerin and streptokinase. Clin Cardiol. 1979;2:354–363. doi: 10.1002/clc.4960020507 121799 10.1002/clc.4960020507

[3] Verheye S Jolicœur EM Behan MW Pettersson T Sainsbury P Hill J Vrolix M Agostoni P Engstrom T Labinaz M. Efficacy of a device to narrow the coronary sinus in refractory angina. N Engl J Med. 2015;372:519–527. doi: 10.1056/NEJMoa1402556 25651246 10.1056/NEJMoa1402556 PMC6647842

[4] Mohl W Gangl C Jusić A Aschacher T De Jonge M Rattay F. PICSO: from myocardial salvage to tissue regeneration. Cardiovasc Revasc Med. 2015;16:36–46. doi: 10.1016/j.carrev.2014.12.004 25616738 10.1016/j.carrev.2014.12.004

[5] Ido A Hasebe N Matsuhashi H Kikuchi K. Coronary sinus occlusion enhances coronary collateral flow and reduces subendocardial ischemia. Am J Physiol Heart Circ Physiol. 2001;280:H1361–H1367. doi: 10.1152/ajpheart.2001.280.3.H1361 11179085 10.1152/ajpheart.2001.280.3.H1361

[6] Knuuti J Wijns W Saraste A Capodanno D Barbato E Funck-Brentano C Prescott E Storey RF Deaton C Cuisset T; ESC Scientific Document Group. 2019 ESC Guidelines for the diagnosis and management of chronic coronary syndromes. Eur Heart J. 2020;41:407–477. doi: 10.1093/eurheartj/ehz425 31504439 10.1093/eurheartj/ehz425

[7] Yeh RW Valsdottir LR Yeh MW Shen C Kramer DB Strom JB Secemsky EA Healy JL Domeier RM Kazi DS. Parachute use to prevent death and major trauma when jumping from aircraft: randomized controlled trial. BMJ. 2018;363:k5094. doi: 10.1136/bmj.k5094 30545967 10.1136/bmj.k5094 PMC6298200

[8] Erlinge D Maehara A Ben-Yehuda O Bøtker HE Maeng M Kjøller-Hansen L Engstrøm T Matsumura M Crowley A Dressler O; PROSPECT II Investigators. Identification of vulnerable plaques and patients by intracoronary near-infrared spectroscopy and ultrasound (PROSPECT II): a prospective natural history study. Lancet. 2021;397:985–995. doi: 10.1016/S0140-6736(21)00249-X 33714389 10.1016/S0140-6736(21)00249-X

[9] Aird WC. Discovery of the cardiovascular system: from Galen to William Harvey. J Thromb Haemost. 2011;9:118–129. doi: 10.1111/j.1538-7836.2011.04312.x 21781247 10.1111/j.1538-7836.2011.04312.x

